# Elisidepsin Interacts Directly with Glycosylceramides in the Plasma Membrane of Tumor Cells to Induce Necrotic Cell Death

**DOI:** 10.1371/journal.pone.0140782

**Published:** 2015-10-16

**Authors:** José Manuel Molina-Guijarro, Carolina García, Álvaro Macías, Luis Francisco García-Fernández, Cristina Moreno, Fernando Reyes, Juan Fernando Martínez-Leal, Rogelio Fernández, Valentín Martínez, Carmen Valenzuela, M. Pilar Lillo, Carlos M. Galmarini

**Affiliations:** 1 Departamento de Investigación y Desarrollo, PharmaMar S.A., Colmenar Viejo, Madrid, Spain; 2 Departamento de Química Física Biológica, Instituto de Química-Física “Rocasolano” (CSIC), Madrid, Spain; 3 Instituto de Investigaciones Biomédicas “Alberto Sols” (CSIC-UAM), Madrid, Spain; 4 Fundación MEDINA, Parque Tecnológico de Ciencias de la Salud, Armilla, Granada, Spain; Toho University School of Medicine, JAPAN

## Abstract

Plasma membrane integrity is essential for cell life. Any major break on it immediately induces the death of the affected cell. Different molecules were described as disrupting this cell structure and thus showing antitumor activity. We have previously defined that elisidepsin (Irvalec^®^, PM02734) inserts and self-organizes in the plasma membrane of tumor cells, inducing a rapid loss of membrane integrity, cell permeabilization and necrotic death. Here we show that, in sensitive HCT-116 colorectal cells, all these effects are consequence of the interaction of elisidepsin with glycosylceramides in the cell membrane. Of note, an elisidepsin-resistant subline (HCT-116-Irv) presented reduced levels of glycosylceramides and no accumulation of elisidepsin in the plasma membrane. Consequently, drug treatment did not induce the characteristic necrotic cell death. Furthermore, GM95, a mutant derivative from B16 mouse melanoma cells lacking ceramide glucosyltransferase (UGCG) activity and thus the synthesis of glycosylceramides, was also resistant to elisidepsin. Over-expression of UGCG gene in these deficient cells restored glycosylceramides synthesis, rendering them sensitive to elisidepsin, at a similar level than parental B16 cells. These results indicate that glycosylceramides act as membrane targets of elisidepsin, facilitating its insertion in the plasma membrane and the subsequent membrane permeabilization that leads to drug-induced cell death. They also indicate that cell membrane lipids are a plausible target for antineoplastic therapy.

## Introduction

The plasma membrane is a biological structure made of hundreds of different lipids arranged in two asymmetric leaflets and a plethora of proteins. It defines the boundary of every living cell and its integrity is essential for life. Plasma membrane not only separates cell contents from the external environment but also regulates what enters and exits the cell, contributes to maintain cell shape and it is involved in different cellular processes such as polarity, adhesion, invasion and motility. Moreover, it is also known that changes in cell membrane composition and structure have important implications in many cancer processes [1]. The particularities of the cell membrane of a malignant tumor cell may influence its ability to grow, attach and respond to neighboring cells differently. It may also affect cancer cell motility, favoring tumor invasion and metastasis.

Given that its integrity is required for survival, plasma membrane constitutes a sort of cellular Achilles heel, sensitive both to mechanical rupture and molecule-driven alterations. A break in the integrity of the plasma membrane immediately compromises its essential role as a barrier, resulting in the death of the affected cell. Not surprisingly, many organisms have developed pore-forming molecules designed to disturb membrane integrity for a variety of purposes. Bacteria and other microorganisms (e.g. parasites) use them to wage war against rival bacteria and to attack human cells [2–4]. Not to be overlooked, our immune cells also produce pore-forming molecules, such as the complement component C9, to attack bacteria and protozoa, and perforin, a protein that kills virus-infected cells [5, 6].

Based on the differences that exist between cell membranes of malignant and normal cells, diverse antitumor molecules were described as targeting this cell structure [1]. In this sense, we have previously described the effects of elisidepsin (Irvalec^®^, PM02734), a synthetic cyclodepsipeptide closely related to the natural product Kahalalide F [7]. Elisidepsin rapidly inserts in the plasma membrane, where it self-organizes and promotes lipid bilayer restructuration [8]. It then induces a rapid loss of membrane integrity and necrotic cell death [8]. Although the sequence of these biological events is well known, the target of elisidepsin at the cell membrane was unnamed. Here, we identify glycosylceramides as the main target of elisidepsin in tumor cell membranes. Moreover, we demonstrate that the absence of this lipid species render tumor cells resistant to elisidepsin. In contrast, reactivation of glycosylceramide synthesis restores elisidepsin sensitivity in the resistant cells. Altogether, these results indicate that glycosylceramides act as elisidepsin targets, in order to trigger the membrane permeabilization that leads to drug-induced cell death. They also suggest that tumor plasma membrane lipids are a valid target for anticancer treatment.

## Materials and Methods

### Reagents

Elisidepsin (C77H125F3N14O18, MW:1591.89) and its two derivatives, Oregon Green^®^ labelled elisidepsin (Irv-OG488) and Alexa Fluor^®^ 555 labelled elisidepsin (Irv-A555), were manufactured at PharmaMar SA. Syringomycin E was purified at PharmarMar. Sulforhodamine B (SRB), Trizma^®^ base, Hoechst-33342, propidium iodide (PI), Dulbecco's modified Eagle's medium (DMEM), McCoy’s medium, penicillin, streptomycin, fetal bovine serum (FBS), thioflavine S, orcinol, sulphuric acid and chloroform HPLC grade were purchased from Sigma (St. Louis, MO, USA). Methanol HPLC grade was from Carlo Erba (Val de Reuil, France). D,L-threo-1-Phenyl-2-decanoylamino-3-morpholino-1-propanol (PDMP) and the neutral glycosphingolipid mixture were from Matreya (Pleasant Gap, PA, USA). C16-β-D-glucosyl ceramide was from Avanti Polar Lipids (Alabaster, AL, USA). n-Butyldeoxynojyrimicin (*N*B-DNJ, Miglustat hydrochloride) was from Tocris Bioscience (Bristol, UK). Cytotoxic compounds were purchased as follows: Amphotericin B, Edelfosine, Paclitaxel, Docetaxel, Epothilone B, Vinorelbine, Vinblastine, Colchicine, Doxorubicin, Cisplatin, Mitomycin C, 5-fluorouracil (5-FU), Irinotecan, Etoposide and Methotrexate were from Sigma; Gemcitabine was from Lilly (Indianapolis, IN, USA); Erlotinib was from Selleckchem (Houston,TX, USA).

### Cell lines and cell culture

Colorectal carcinoma HCT-116 (CCL-247) cell line used in this study was purchased from the American Type Culture Collection. The mouse melanoma cell line B16 (RCB0557) and the mutant derivative GM95 (RCB1026) were purchased from the Riken Cell Bank (Ibaraki, Japan). The elisidepsin resistant cell line HCT-116-Irv was developed in our laboratory from the HCT-116cell line, using the classical stepwise selection method [9, 10]: the parental cells were treated with increasing concentrations of elisidepsin and, at each stage, a stable surviving population of cells was stablished before the next increment. The maximal elisidepsin concentration used during the selection period was 100 μM and the overall process lasted 18 months. HCT-116 and HCT-116-Irv were cultured in McCoy’s medium supplemented with 10% FBS, 2 mM glutamine and 100 U/ml penicillin and streptomycin at 37°C and 5% CO_2_. B16 and GM95 cells were maintained in DMEM with the same supplements and conditions.

### Cell viability assays

Cells were seeded in 96-well microtiter plates and maintained at 37°C and 5% CO_2_ in drug-free medium before treatment with vehicle alone or elisidepsin at the concentrations and times indicated in the text. For cell survival quantification, a colorimetric assay using SRB was used [11]. Briefly, after treatment, cells were washed twice with phosphate buffered saline (PBS), fixed for 25 min in 1% glutaraldehyde solution, rinsed twice with PBS, stained in 0.4% SRB-1% acetic acid solution for 30 min, rinsed several times with 1% acetic acid solution, and air-dried. SRB was then extracted in 10 mM Trizma base solution, and the absorbance was measured at 490 nm in a microplate spectrophotometer. Results are expressed as percentage of control cell survival and represent the mean of at least three independent experiments. IC_50_, half-maximal inhibitory concentration, was used as a reference value.

### Quantification of elisidepsin accumulation in tumor cells

HCT-116 cells were treated in triplicate with elisidepsin 7 μM in P6 culture plates at the times indicated in the text. After treatment, cells were washed twice with cold PBS and scraped in the same buffer. Cells were centrifuged and resuspended in 100 μL of methanol to extract the compound bound to cells. Other plates were treated in the same conditions and the cells were collected to determine the protein content in each case. The quantity of elisidepsin in each sample was determined by HPLC-MS. The analysis consisted of gradient reversed phase chromatography followed by positive ion electrospray tandem mass spectrometry (ESI/MS/MS) detection using multiple reactions monitoring (MRM). The analytical column (Waters, YMC C18 3 μm, 50 x 2.1 mm) was placed in an oven at 50°C. The flow rate was 600 μL/min. The wash solvent 1 & 2 used was acetonitrile:2-propanol:methanol:water (1:1:1:1). Mobile phase A was water:formic acid (500:0.5). Mobile phase B was acetonitrile:formic acid (500:0.5). The equipment used was composed of a binary 10 AD vp HPLC (Shimadzu, Kyoto, Japan), a controller (Shimadzu), an oven (Shimadzu), an autosampler (CTC PAL, Leap Technologies; Carrboro, NC, USA) and a triple quadropole mass spectrometer (API4000, AB Sciex, Framingham, MA, USA). Data were analyzed with Software Analyst version 1.5 with intelliquan quantitation tool (AB Sciex).

### Membrane permeabilization assays

Cells were seeded in P24 plates and cultured for 48 h as described above. Then, medium was replaced with fresh culture medium (supplemented with 25 mM HEPES pH 7.4 and 50 μg/mL PI) containing or not different concentrations of elisidepsin. Morphological changes and nuclei staining in membrane permeabilized cells were followed by phase contrast and fluorescence microscopy.

### Electrophysiological recordings

The effects of elisidepsin on membrane conductance were analyzed in HCT-116 and HCT-116-Irv cells as previously described [12–15]. Experiments were performed in a small bath mounted on the stage of an inverted microscope (Nikon model TMS, Garden City, NY, USA) continuously perfused with the extracellular solution (Tyrode-glucose buffer). Ion currents were recorded at room temperature (20–22°C) using the whole-cell voltage-clamp configuration of the patch-clamp technique [16] with an Axopatch 200B patch-clamp amplifier (Axon Instruments, Foster City, CA). Currents were filtered at 2 kHz (four-pole Bessel filter), sampled at 4 kHz. Data acquisition and command potentials were controlled by the CLAMPEX program of PCLAMP 9.2 software (Axon Instruments). Micropipettes were pulled from borosilicate glass capillary tubes (Narishige, GD-1, Tokyo, Japan) on a programmable horizontal puller (Sutter Instrument Co., San Rafael, CA, USA) and heat-polished with a microforge (Narishige). Pipette tip resistance averaged between 1 and 3 MΩ. The intracellular pipette solution contained (in mM): K-aspartate 80, KCl 50, phosphocreatine 3, KH_2_PO_4_ 10, MgATP 3, HEPES-K 10, EGTA-K 5 and was adjusted to pH 7.25 with KOH. The external solution (Tyrode-glucose buffer) contained (in mM): NaCl 130, KCl 4, CaCl_2_ 1.8, MgCl_2_ 1, HEPES-Na 10, and glucose 10, and was adjusted to pH 7.40 with NaOH. Measurements were performed using the CLAMPFIT program of PCLAMP 9.2.

### FLIM-FRET-phasor approach

Two-photon fluorescence-lifetime imaging microscopy (FLIM) of live cells was carried out on a MicroTime 200 system (PicoQuant, Germany) coupled with an Olympus IX71 inverted microscope described in detail elsewhere [8]. In short, excitation at 755 nm was performed by a mode-locked, femtosecond-pulsed Ti:Sapphire laser (Mai-Tai, Spectra Physics, CA). Two-color fluorescence images were acquired simultaneously with two single-photon avalanche diodes (SPAD, SPCM-AQR-14, Perkin Elmer, USA), through a dichroic beam splitter FF560-Di01 and bandpass filters FF01-520/35 (Donor FRET channel: Irv-OG488), FF01-685/40 (Acceptor FRET channel: Irv-A555) from Semrock, Germany. Acquisition time per pixel accounted for 1.2–2 ms.

FLIM-FRET (Föster Resonance Energy Transfer) measurements: HCT-116 and HCT-116-Irv cells were cultured in poly-L-lysine coated LabTek-II chambered coverglass slides (Thermo Scientific-Nunc). The coating method consists in incubation of slides with poly-L-Lysine 0.1 mg mL^-1^ in sterile PBS, overnight at 4°C, followed by three washes with PBS. After 48 h culture, cells were washed with Tyrode-glucose buffer and treated with a mix of FRET donor (Irv-OG488) 200 nM, FRET acceptor (Irv-A555) 800 nM and elisidepsin 3 μM, keeping DMSO lower than 0.5% v/v.

FLIM-FRET provides an intensity independent measurement of FRET. The fluorescence lifetime is defined by the average time the fluorophore spends in the excited state, before to return to the ground state, and it is characteristic of each fluorophore and its microenvironment. Phasor analysis transforms the fluorescence lifetime data from each pixel into a coordinate pair corresponding to the phase (Φ) and modulus (M) of a vector (phasor) [17], which is represented as a point with coordinates (*s*,*g*) in the phasor plot. In a reciprocal manner, each point in the phasor plot can be mapped to a pixel of the FLIM image, facilitating the identification of molecular species in a graphical way, avoiding fitting of multiexponential functions that would require a large number of photons per pixel, which in general it is not possible to accumulate in living cell measurements. Clusters of pixels with similar phasors in specific regions of the phasor plot, selected by circular color cursors, are assigned to specific molecular species: donor only, FRET and autofluorescence background.

When donor and acceptor molecules are at FRET distances (lower than 100 Å), the fluorescence lifetime of the donor would decrease from *τ*_*D*_ (donor unquenched) to *τ*_*DA*_ (donor quenched by the presence of the acceptor) as a function of the FRET efficiency (*E*): *τ*_*DA*_ = *τ*_*D*_ *·(1-E)*. FRET trajectories on the phasor plot are curved and they would represent realizations of all possible donor phasors quenched by FRET with different efficiencies. FRET efficiencies were estimated using the FRET calculator tool included in SimFCS program (Laboratory for Fluorescence Dynamics, Irvine, CA), taking in account the contribution of donor only and background lifetime species present in each pixel.

### Cell lipids extraction and analysis

Lipid extracts from cells were obtained according to the Bligh and Dyer method with slight modifications [18]. Briefly, a cell pellet was resuspended in 1 mL of distilled water, then 3.75 mL of chloroform:methanol (1:2) was added and the suspension was mixed by vortexing two minutes; next, 1.25 mL of chloroform was added and vortexed one minute; last, 1.25 mL of NaCl 1 M was added to the suspension and vortexed one minute. The suspension was centrifuged and the bottom organic phase was collected. This solution was dried under a nitrogen stream and stored at -20°C. This extract was dissolved in a small volume of chloroform:methanol (2:1) and lipids were separated in a HPTLC (high performance thin layer chromatography) silica gel plate (Merck-Millipore, Darmstad, Germany) using a mobile phase of chloroform:methanol (8:2). Lipids were visualized by staining the plate with thioflavine S (sprayed with 10 μg mL^-1^ in acetone:water (4:1) and visualized under UV light). Glycosylated lipids were visualized by orcinol staining (sprayed with orcinol 0.5% in sulphuric acid 0.5 M and developed with heat). Lipids were purified from thioflavine S stained plates by scratching the resin followed by extraction with chloroform:methanol (2:1).

### Interaction of elisidepsin with lipids

Interaction of purified lipid fractions with elisidepsin was evaluated with an overlay assay. Lipids were applied onto a nitrocellulose membrane (Protran membrane, Whatman-GE, Piscataway, NJ, USA), blocked with non-fat milk 1% in PBS, and hybridized with a biotinilated elisidepsin derivative or biotin as control (6 μM in blocking solution, 1 h at room temperature with agitation). Elisidepsin-biotin interactions were detected by incubation with streptavidin-HRP and subsequent chemiluminescence.

### Overexpression of UGCG

GM95 cell line was transfected with an expression vector containing the GFP-tagged UGCG human cDNA (Origene). For the transfection, Lipofectamine reagent (Life Technologies) was used according the manufacturer’s protocol. Positive clones were selected with G-418 and by fluorescence intensity.

### NMR analysis of purified lipids

A purified lipid fraction obtained from HCT-116 cell lipids was analyzed to determine its NMR spectrum. ^1^H NMR was recorded on a Varian Unity 500 spectrometer (Palo Alto, CA, USA) at 500 MHz. Chemical Shifts (δ) are reported in parts per millions (ppm) referenced to CH_3_OH at 3.30 ppm.

## Results

### Characterization of elisidepsin resistance in HCT-116-Irv cells

**Fig 1:**
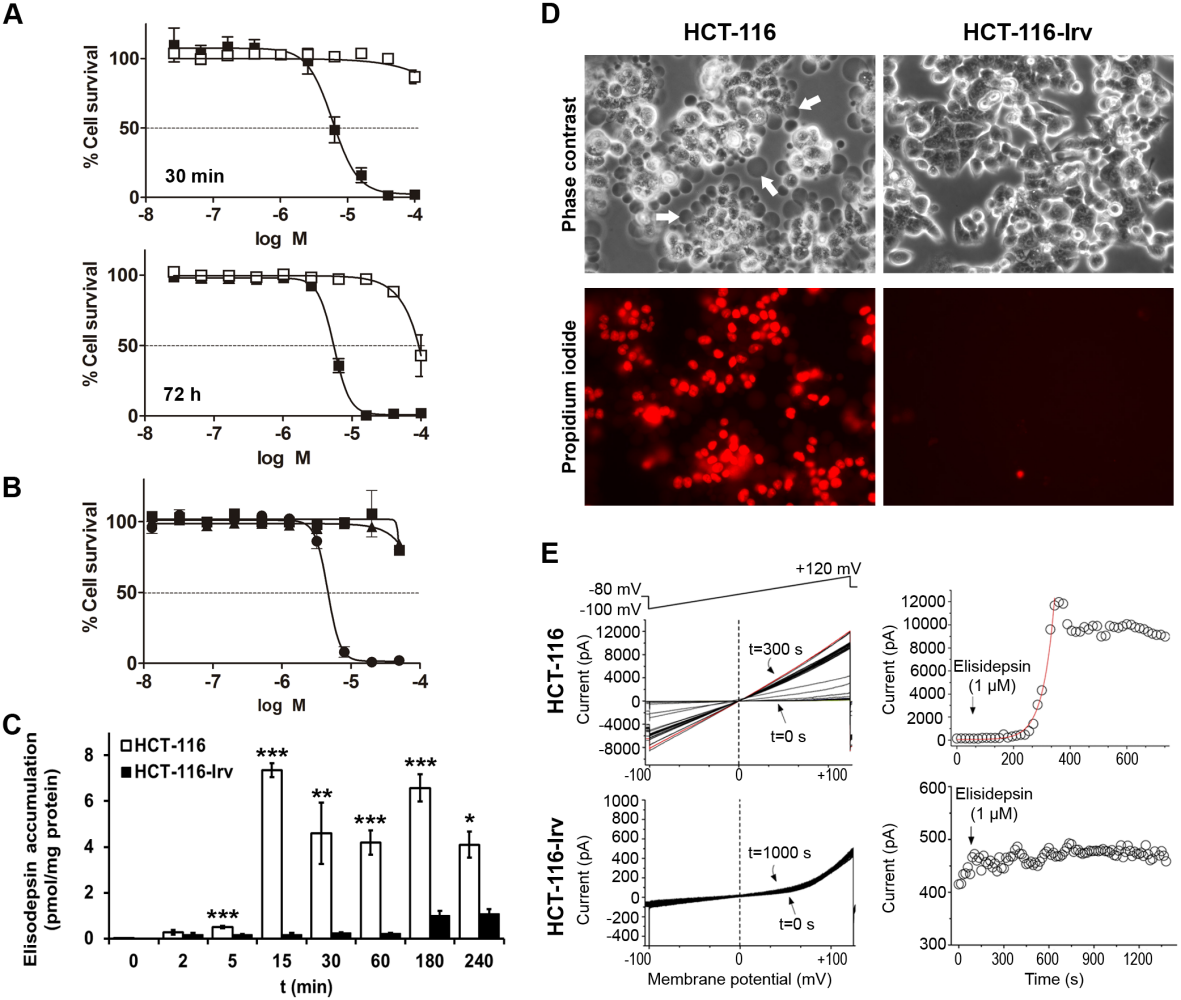
Characterization of the HCT-116-Irv resistance to elisidepsin. (**A**) Concentration-response curves showing the activity of elisidepsin after 30 min and 72 h in HCT-116 (⬛) and HCT-116-Irv cells (⬜); results represent the mean±SD of at least three different experiments. (**B**) Stability of elisidepsin resistance in HCT-116-Irv cells determined by concentration-response curves after 2 (⬛) and 15 (▲) culture passages in absence of the compound. HCT-116 cells (●) are also depicted in the graph. Results represent the mean±SD of three different experiments. (**C**) Elisidepsin accumulation (pmol/mg protein) in HCT-116 (white bars) and HCT-116-Irv cells (black bars). Both cell lines were treated with elisidepsin 7 μM at different time points and the accumulated compound was quantified by HPLC/MS. Results represent the mean±SD of three different samples. Comparisons between different samples were analyzed by Student’s *t* test. Differences were considered significant at *P<0.05, **P<0.01 and ***P<0.001. (**D**) Representative images of HCT-116 and HCT-116-Irv cells exposed to elisidepsin 10 μM for 5 min. Phase contrast microscopy images show morphological alterations and giant vesicles formation only in the wild-type cells (white arrows). Fluorescence microscopy shows PI stained nuclei only in the parental cells. (**E**) Electrophysiological effects of elisidepsin 1 μM on HCT-116 and HCT-116-Irv cells. Left panels show original records after applying a ramp pulse protocol from -100 mV to +120 mV during 500 ms. Right panels show the amplitude of the maximum current at the end of the ramp together with the exponential fit of the process. HCT-116-Irv cells are completely insensitive to the elisidepsin effects as shown in the current records. Holding potential was maintained at -80 mV.

HCT-116-Irv cells were derived from HCT-116 parental cells by a classical stepwise selection procedure during 18 months. After this period, a pool of resistant cells with a very homogenous behavior in all the analyzed variables was stablished and it was consider as a new cell line named HCT-116-Irv. As shown in Fig 1A and Table 1, cells were 14.7-fold more resistant to elisidepsin than parental HCT-116 cells. The IC_50_ values after 30 min exposure were 7.7±4.1 μM and >100 μM μM for HCT-116 and HCT-116-Irv cells, respectively (Fig 1A). The IC_50_ values after 72 h exposure were 5.5±0.8 μM and 81.5±0.8 μM for HCT-116 and HCT-116-Irv cells, respectively (Fig 1A). To determine the stability of the acquired resistance in HCT-116-Irv, cells were cultured for 15 passages in the absence of the drug and concentration-response curves were performed in passage 2 and 15. The levels of resistance at both time points were very similar, indicating that HCT-116-Irv cells had acquired a permanent resistance to the drug (Fig 1B). HCT-116-Irv cell line did not show any cross resistance with other common anticancer agents or other agents interacting with the cell membrane, indicating that the resistance mechanism was specific for elisidepsin (Table 1). Additionally, we evaluated the accumulation of the compound in both parental and resistant cells. Both cell lines were treated with elisidepsin 7 μM at several time points, and the amount of drug retained in cells was quantified by HPLC/MS. While the parental cells showed a rapid and large accumulation of elisidepsin, in the resistant cells the accumulation of the compound was remarkably smaller and slower (Fig 1C).

**Table 1:** Antiproliferative activity of elisidepsin and other compounds in HCT-116 and HCT-116-Irv tumor cell lines.

Compound	HCT-116 (IC_50_)^a^	HCT-116-Irv (IC_50_)^a^	RR^b^
**Elisidepsin**	5.5 x 10^−6^ M	8.1 x 10^−5^ M	14.7
**Amphotericin B**	5.2 x 10^−6^ M	6.1 x 10^−6^ M	1.2
**Syringomycin E**	2.0 x 10^−5^ M	1.5 x 10^−5^ M	-1.4
**Edelfosine**	2.9 x 10^−5^ M	2.9 x 10^−5^ M	1.0
**Paclitaxel**	6.9 x 10^−9^ M	8.3 x 10^−9^ M	1.2
**Docetaxel**	2.9 x 10^−9^ M	1.8 x 10^−9^ M	-1.6
**Epothilone B**	2.2 x 10^−9^ M	3.9 x 10^−9^ M	1.8
**Vinorelbine**	1.6 x 10^−7^ M	1.9 x 10^−7^ M	1.1
**Vinblastine**	3.2 x 10^−10^ M	4.1 x 10^−10^ M	1.3
**Colchicine**	3.9 x 10^−9^ M	2.8 x 10^−9^ M	-1.4
**Doxorubicin**	1.4 x 10^−7^ M	9.9 x 10^−8^ M	-1.4
**Cisplatin**	9.8 x 10^−6^ M	3.6 x 10^−6^ M	-2.7
**Mitomycin C**	3.3 x 10^−7^ M	3.0 x 10^−7^ M	-1.1
**5-FU**	4.0 x 10^−6^ M	1.1 x 10^−5^ M	2.8
**Irinotecan**	2.5 x 10^−6^ M	3.1 x 10^−6^ M	1.2
**Gemcitabine**	6.3 x 10^−10^ M	6.7 x 10^−10^ M	1.1
**Etoposide**	1.2 x 10^−6^ M	9.6 x 10^−7^ M	-1.2
**Methotrexate**	3.7 x 10^−8^ M	7.7 x 10^−8^ M	2.1
**Erlotinib**	3.2 x 10^−5^ M	1.6 x 10^−5^ M	-2.0

^a^ IC_50_, cell growth half-maximal inhibitory concentration. Values represent the mean of three different experiments.

^b^ RR (resistance ratio): fold change between IC_50_ values from HCT-116-Irv and HCT-116

### Typical cell membrane perturbations caused by elisidepsin are not observed in HCT-116-Irv cells

We have previously demonstrated that elisidepsin molecules localize in the cell membrane, close enough to each other as to suggest that the compound could self-organize, forming supramolecular structures that likely trigger necrosis through the disruption of membrane integrity [8]. We thus evaluated whether exposure to elisidepsin induced similar phenomena in HCT-116-Irv cells. For this purpose, parental and elisidepsin-resistant cells were cultured in the presence of propidium iodide (PI) and exposed to the drug (10 μM) for 5 min. Cells were observed by fluorescence microscopy. As shown in Fig 1D and S1 Movie, most of HCT-116 cells exhibited a nuclear staining due to the uptake of PI. Simultaneously, cell morphology was seriously altered with the formation of giant vesicles. All these effects were a consequence of the membrane damage caused by elisidepsin. In contrast, no PI uptake or giant vesicle formation was detected in the resistant cells (Fig 1D).

We finally analyzed the effect of the non-toxic concentration of elisidepsin 1 μM on membrane conductivity in HCT-116 and HCT-116-Irv cells using a series of ramps from -100 mV to +120 mV during 500 ms (Fig 1E). Right upper panel of Fig 1E shows the time course of the elisidepsin effects on HCT-116 cells. As it is shown in the left upper panel, elisidepsin produced an increase of the membrane conductance at all membrane potentials (negative and positive) that lead to the appearance of an inward and an outward current at negative and positive membrane potentials, respectively. The time constant indicated a fast process (107±39 s; n = 4). The latency between the application of elisidepsin and the beginning of the effects was 460±163 s (n = 4). The compound had virtually no effect in HCT-116-Irv cells (Fig 1E, lower panels).

### Elisidepsin inserts differently in the cell membrane of sensitive and resistant HCT-116 cells

We have used the FLIM-FRET (Fluorescence Lifetime Imaging-Föster Resonance Energy Transfer) phasor approach to investigate whether elisidepsin inserts in a similar way into the plasma membrane of HCT-116 and HCT-116-Irv cells. In this approach the fluorescence decay from each pixel of the image is represented as a point in the phasor plot. This method improves the Fast FLIM-FRET approach used in a previous work [8], and provides a simple and quantitative graphical way of comparison of the two types of cells in terms of donor-unquenched and donor-acceptor lifetimes and FRET efficiencies. Irv-OG488 (elisidepsin-Oregon Green 488) and Irv-A555 (elisidepsin-Alexa Fluor 555) are respectively, the donor and the acceptor molecules in the FRET assay.

**Fig 2:**
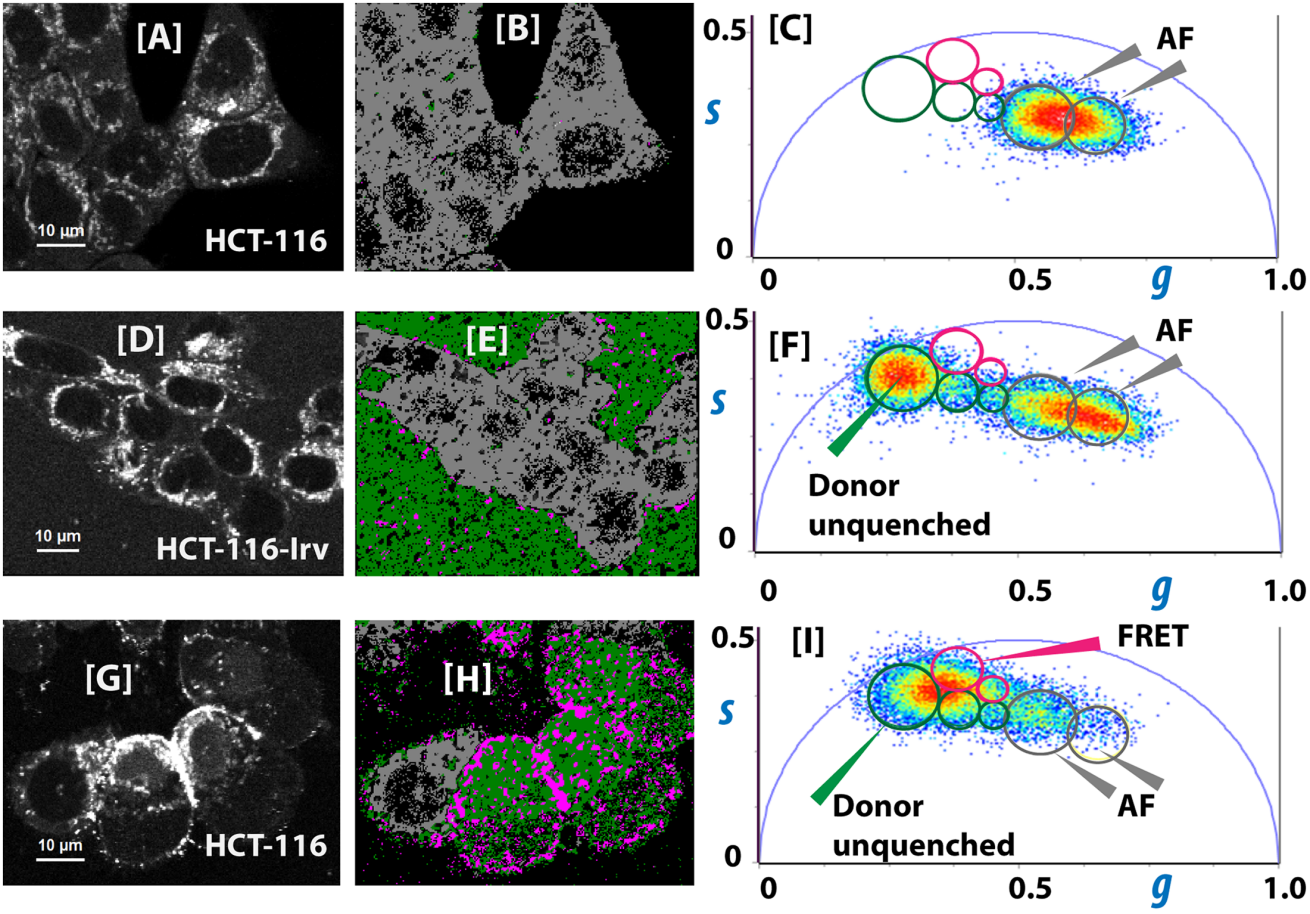
Comparative interaction study of elisidepsin and the plasma membrane of HCT-116 and HCT-116-Irv cells. Untreated HCT-116 cells: Autofluorescence intensity image **(A)**, FLIM-phasor image **(B)** and the corresponding phasor plot **(C)** of representative untreated HCT-116 cells. Grey mask on the FLIM-phasor image, and grey circles on phasor plot correspond to autofluorescence phasors. HCT-116-Irv cells treated with 5μM total elisidepsin (FRET donor: Irv-OG488 200 nM, FRET acceptor: Irv-A555 800 nM, elisidepsin 3 μM, 1:4:15): Fluorescence intensity image **(D)**, FLIM-phasor image **(E)**, and the corresponding phasor plot **(F)** of representative elisidepsin treated HCT-116-Irv cells. Green mask on FLIM-phasor image and green circles on phasor plot correspond to FRET donor (Irv-OG488) molecular species outside the cell where donor and acceptor molecules are at distances longer than 70 Å. For these species, the lifetime of Irv-OG488 in the media is not affected by the presence of Irv-A555 molecules, and it was assigned to the donor-only (*τ*_*D*_, donor unquenched) phasor in the phasor plot for FLIM-FRET-phasor analysis. The lifetimes inside the cells have not changed substantially with respect to untreated cells, showing the typical autofluorescence behavior (grey mask and cursor). Elisidepsin molecules were not detected inside HCT-116-Irv treated cells. HCT-116 cells treated with 4μM total elisidepsin (FRET donor: Irv-OG488 200 nM, FRET acceptor: Irv-A555 800 nM, elisidepsin 3 μM, 1:4:15): Fluorescence intensity image **(G)**, FLIM-phasor image **(H)**, and the corresponding phasor plot **(I)** of representative elisidepsin treated HCT-116 cells. Green mask on FLIM-phasor image and green circles on phasor plot correspond to Irv-OG488 molecular species inside the cell that are not complexed with other elisidepsin molecules, so do not change their lifetime (donor unquenched lifetime). Pink mask and pink cursor correspond to elisidepsin complexes, containing elisidepsin, Irv-OG488 and Irv-A555 molecules at distances shorter than 50 Å, mainly located in the plasma membrane of affected cells. The lifetime of the FRET donor (Irv-OG488) in these complexes (*τ*_*DA*_) decreases due to FRET to neighboring FRET acceptor (Irv-A555) molecules, and their corresponding phasor fall in the quenching FRET trajectory. Excitation wavelength: 755 nm. Laser frequency (*f*): 80x10^6^ s^-1^. Emission: FF01 520/35. Phasor plot: ***g*** and ***s*** axis represent the real and imaginary parts of the Fourier transform of the fluorescence impulse response *I(t)*: $g\left(\omega \right)\;=\;\int_{0}^{\infty }I\left(t\right)\cdot \text{cos}\omega t\cdot dt\;=\;M\cdot \text{cos}\phi$; $s\left(\omega \right)\;=\;\int_{0}^{\infty }I\left(t\right)\cdot \text{sin}\omega t\cdot dt\;=\;M\cdot \text{sin}\phi$; ω = 2 *π f; M* and *ϕ* are respectively the modulus and the phase of the phasor.

Fig 2A shows the autofluorescence intensity image of representative untreated HCT-116 cells, measured in the donor channel, in the same experimental conditions as the FRET samples. Fig 2B shows the reciprocal selection of the grey autofluorescence cursors from the phasor plot of the same cells, represented in Fig 2C.

Fig 2D shows the fluorescence intensity image of representative HCT-116-Irv cells treated with 4 μM total elisidepsin (Irv-OG488 200 nM, Irv-A555 800 nM, elisidepsin 3 μM, 1:4:15). We observe a low fluorescence signal in the media, with a fluorescent lifetime characteristic of Irv-OG488 molecules, represented by the green mask and the green cursor respectively in the FLIM-phasor image (Fig 2E), and the phasor plot (Fig 2F). This result indicates that most of the Irv-OG488 molecules remained in the media, far from Irv-A555 molecules, and did not interact with HCT-116-Irv cells. Inside the cell, there were not significant differences in the autofluorescence of treated and untreated cells, showing grey color masks corresponding to grey cursors in the phasor plot (Fig 2F).

Fig 2G shows the fluorescence intensity image of representative HCT-116 cells treated with 4 μM total elisidepsin (Irv-OG488 200 nM, Irv-A555 800 nM, elisidepsin 3 μM, 1:4:15). We observe an important increase in the fluorescence intensity of Irv-OG488 localized mainly in the plasma membrane. The FLIM-phasor image (Fig 2H) shows Irv-OG488 molecules in the plasma membrane and inside HCT-116 affected cells (green mask), with a fluorescence lifetime characteristic of Irv-OG488 (green cursor in the phasor plot; Fig 2I). In addition, we have detected a new Irv-OG488 molecular species in specific regions of the plasma membrane of HCT-116 treated cells, with a fluorescence lifetime shorter than measured for Irv-OG488 (pink cursor in the phasor plot; Fig 2I). The decrease in the fluorescence lifetime is indicative of a FRET process between the FRET donor (Irv-OG488) to the FRET acceptor (Irv-A555) molecules. The distribution of Irv-OG488, Irv-A555, and elisidepsin molecules in the membrane elisidepsin assemblies proves to be non-random. Regions with shorter lifetime would correspond to [elisidepsin]_n_ assemblies in the plasma membrane, containing Irv-OG488 and Irv-A555 molecules at FRET distances (∼60% FRET efficiency), while membrane regions with high Irv-OG488 intensity and unquenched lifetime would correspond to [elisidepsin]_n_ which do not contain Irv-A555 molecules, or containing Irv-OG488 and Irv-A555 molecules far from FRET distances (>100 Å).

In previous FRET studies, using the same fluorescent elisidepsin analogs, we have observed that, when a mix of Irv-OG488 and elisidepsin molecules interacted with the cell membrane of A549 cells, they changed their conformation, forming some kind of assembly in the membrane so that the fluorescence signal per membrane pixel increases several orders of magnitude, compared to the fluorescence detected from Irv-OG488 molecules in solution [8].

These results demonstrated that like A549 cells, at cytotoxic concentrations, elisidepsin molecules are forming some kind of assemblies all throughout the plasma membrane of HCT-116 cells, former to the disruption of membrane integrity. These elisidepsin assemblies were not observed in elisidepsin treated HCT-116-Irv cells.

### Elisidepsin interacts with glycosylated ceramides in the plasma cell membrane

**Fig 3:**
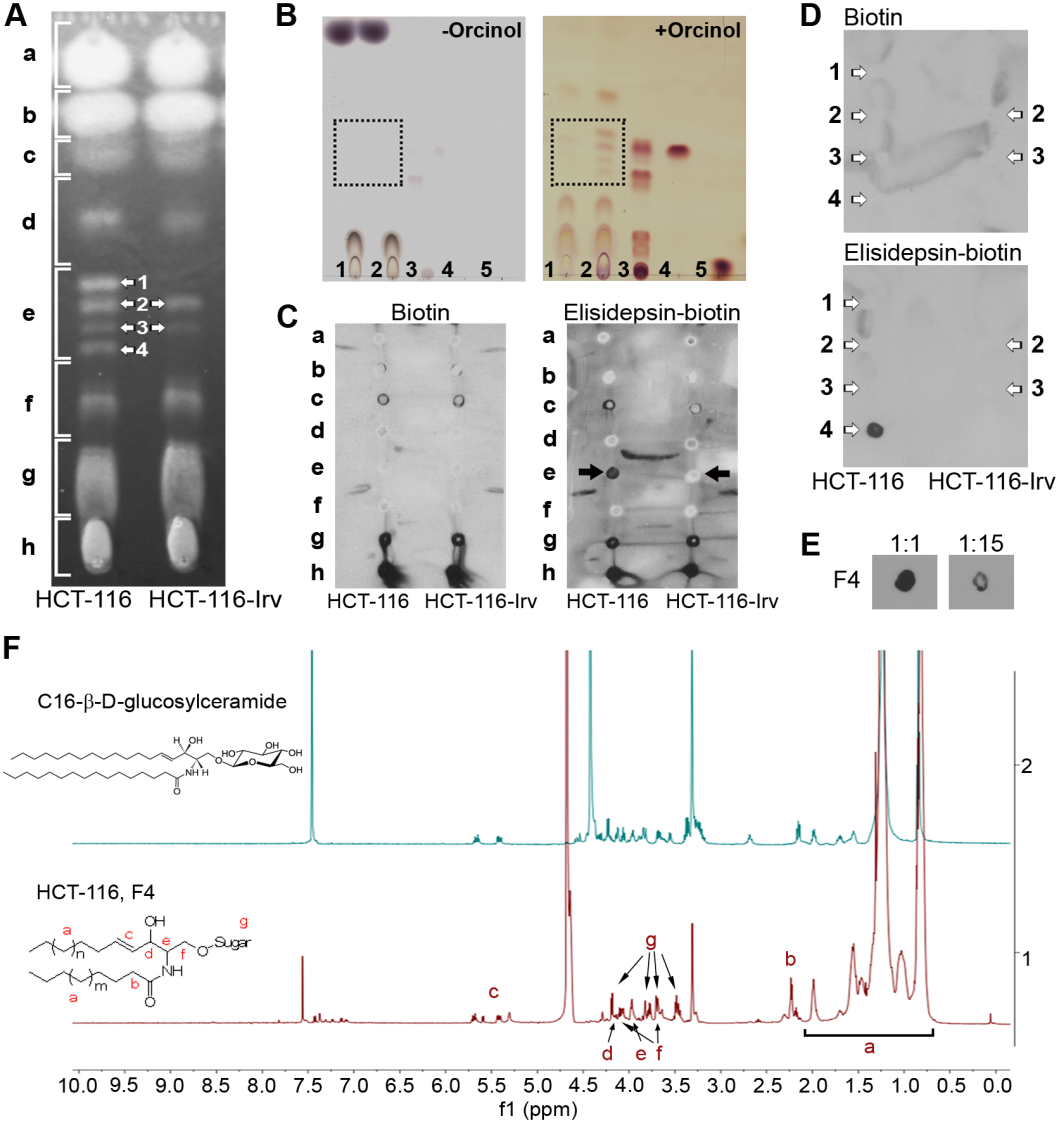
Glycosylceramides pattern and elisidepsin interaction with glycosylceramides in HCT-116 and HCT-116-Irv cells. (**A**) Monodimensional silica-gel HPTLC of the lipid extract from HCT-116 and HCT-116-Irv. The plate was stained with thioflavine S. Arrows show a group of nearby fractions with different presence in both cell lines. The total extract was divided in 8 fractions (a to h) that were purified for subsequent experiments. (**B**) Glycolipid detection in lipid extracts from HCT-116 and HCT-116-Irv. Total lipid extracts from HCT-116 and HCT-116-Irv were developed on HPTLC silica plates and visualized with orcinol-sulphuric acid staining to reveal the presence of glycosylated lipids. Control samples were added to confirm the results; left panel: control HPTLC incubated with sulphuric acid alone; right panel: HPTLC stained with orcinol-sulphuric acid. The samples were: 1, HCT-116-Irv lipid extract (200 μg); 2, HCT-116 lipid extract (200 μg); 3, neutral glycosphingolipid mixture (cerebrosides, lactosylceramides, ceramide trihexosides, globosides –Gb4-) (10 μg); 4, C16-β-D-glucosyl ceramide (6 μg); 5, glucose (5 μg). The lipid fractions that are related to elisidepsin binding or resistance are pointed out inside the dot line square. (**C**) Dot-blot assay for the interaction of biotin or elisidepsin-biotin with the lipid fractions from the wt and the resistant cells. Arrows indicate the fraction with specific binding to elisidepsin-biotin only present in HCT-116 cells. (**D**) Dot-blot assay for the interaction of biotin or elisidepsin-biotin with the selected lipid fractions from the parental and the resistant cells. Fraction 4 from HCT-116 is the only fraction that interacts with elisidepsin-biotin. (**E**) Competitive binding assay with elisidepsin. Nitrocellulose membranes with spots of the lipid fraction 4 from HCT-116 were incubated with different proportions of elisidepsin-biotin and elisidepsin (1:1, left; 1:15, right). A lower signal was detected when elisidepsin quantity was increased. (**F**) NMR analysis of a purified lipid fraction of HCT-116 cells. NMR spectra from C16-β-D-glucosyl ceramide and purified F4 lipid fraction from HCT-116 are shown. Letters and arrows indicate the assignation of signals from F4 spectra in a model glycosylceramide molecule.

Since HCT-116-Irv resistant cells accumulated less elisidepsin and the compound was detected at lower levels in their cell membrane, we investigated whether this was due to an altered lipid composition. To this end, we extracted total lipids from HCT-116 and HCT-116-Irv cell pellets using standard methods based on chloroform and methanol combinations. Lipid extracts were then fractionated by HPTLC (high performance thin layer chromatography) and the plates were stained with specific dyes for lipids (Fig 3A). Interestingly, we identified two subfractions in HCT-116 cells that were almost absent in their resistant counterpart (white arrows 1 and 4 in Fig 3A). Staining HPTLC plates with orcinol, a classic sugar staining method, demonstrated that the two differential lipid species identified contained glycosylated lipids (Fig 3B).

To determine if elisidepsin was interacting specifically with any of the lipid fractions detected in both cell lines, an overlay assay was carried out. The pattern of the total lipid extract was divided in 8 fractions that were collected from a monodimensional HPTLC and named from “a” to “h” (Fig 3A). These lipid fractions were applied on a nitrocellulose membrane and were incubated with an elisidepsin-biotin derivative that facilitated its subsequent detection (Fig 3C). The fraction which contained the differential subfractions identified in the previous experiment, and now called “e”, was positive for the binding to elisidepsin-biotin. This binding occurred only in the extract from the HCT-116 cells. There was no positive signal in the resistant cells extract.

Next, we investigated which of the previously identified differential subfractions was necessary for the binding to elisidepsin-biotin. For this purpose, we performed two preparative HPTLC applying 10 mg of the lipid extracts from each cell line, and the 4 fractions depicted in “e” in Fig 3A were purified separately. Each individual subfraction was assayed again through an overlay assay with elisidepsin-biotin. As seen in Fig 3D, fraction number 4 was identified as the only responsible for the binding to elisidepsin-biotin in parental HCT-116 cells. A competition assay between elisidepsin-biotin and elisidepsin (Fig 3E) showed that elisidepsin could displace the biotinylated compound, indicating the specificity of the binding. An NMR (nuclear magnetic resonance) study of the purified lipid fraction number 4 revealed similarities with a commercial C16-β-D-glucosyl ceramide (Fig 3F).

### Glycosylceramide levels determine the sensitivity of tumor cells to elisidepsin

**Fig 4:**
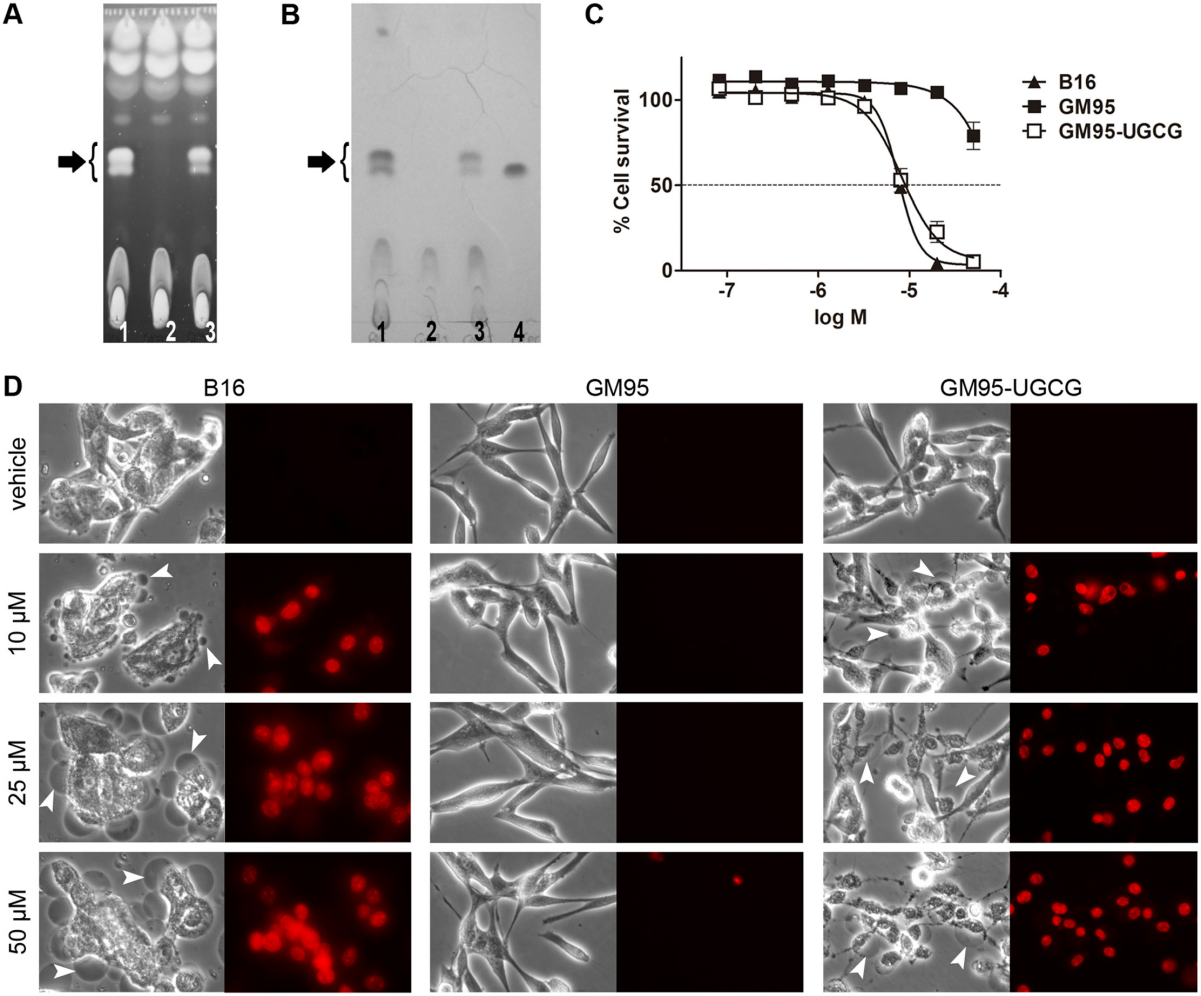
Activity of elisidepsin in B16, GM95 and GM95-UGCG cell lines. (**A**) HPTLC analysis of the total lipid extracts from the three cell lines. Lipids were separated in chloroform:methanol (8:2) and stained with thioflavine S. (**B**) Orcinol staining of glycolipids present in the lipid extracts from the three cell lines. Lipids were separated as mentioned before. In the last lane, C16-β-D-glucosyl ceramide was included as control. In (A) and (B): 1, lipid extract from B16 (200 μg); 2, lipid extract from GM95 (200 μg); 3, lipid extract from GM95-UGCG (200 μg); 4, C16-β-D-glucosyl ceramide (6 μg). Arrows indicate the lipid fractions that are present or completely absent in the cell lines (**C**) Concentration-response curves for elisidepsin obtained after 72 h treatment in B16 (▲), GM95 (⬛) and GM95-UGCG cells (⬜); results represent the mean±SD of at least three different experiments. (**D**) Representative images of B16, GM95 and GM95-UGCG cells exposed to different concentrations of elisidepsin (10, 25 and 50 μM) for 5 min. Phase contrast microscopy images show morphological alterations and giant vesicles formation only in B16 and GM95-UGCG cells (white arrows). PI stained nuclei are shown in the fluorescence microscopy images from both cell lines. GM95 cells do not show any response to the treatment with elisidepsin.

**Fig 5:**
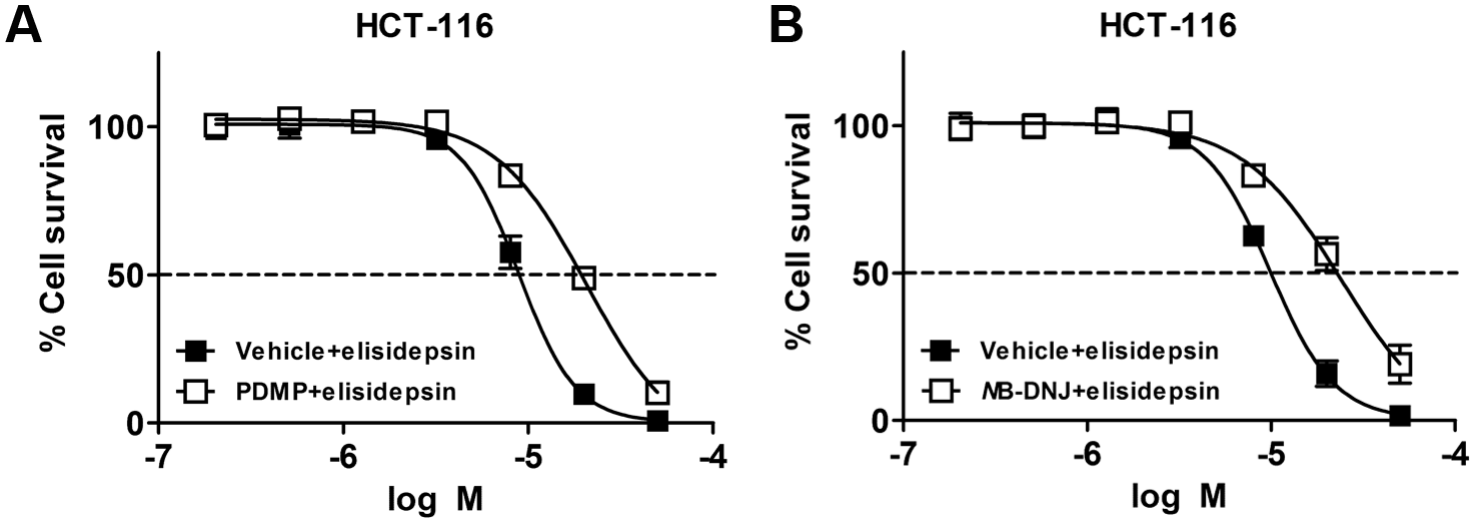
Effect of glucosylceramide synthase inhibitors, PDMP and *N*B-DNJ on the antiproliferative activity of elisidepsin. (**A**) Concentration-response curves of elisidepsin (30 min incubation) in HCT-116 cells pretreated for 72 h with the glucosylceramide synthase inhibitor PDMP 10 μM (⬜) or vehicle (⬛); (**B**) Concentration-response curves of elisidepsin (30 min incubation) in HCT-116 cells pretreated for 48 h with the glucosylceramide synthase inhibitor *N*B-DNJ 200 μM (⬜) or vehicle (⬛). Results represent the mean±SD of three different experiments.

We assessed if glycosylceramides were needed for elisidepsin-induced disruption of the plasma membrane and necrosis. For this purpose we used the mouse melanoma B16 cell line and its mutant derivative GM95, described to lack UDP-glucose:ceramide glucosyltransferase (UGCG; EC 2.4.1.80) activity [19]. HPTLC analysis of lipid extracts from both cell lines confirmed the absence in GM95 cells of lipids in the region of the identified glycosylceramides (Fig 4A and 4B). B16 cells were sensitive to elisidepsin, displaying the typical membrane permeabilization and formation of large vesicles observed in other tumor cell lines (Fig 4C and 4D; Table 2). In contrast, UGCG-deficient GM95 cells were resistant to elisidepsin (Fig 4C and Table 2) showing a behavior similar to that of HCT-116-Irv cells (Fig 4D). Interestingly, over-expression of UGCG gene in GM95 cells (GM95-UGCG cells) rendered them sensitive to elisidepsin, at a similar level of that of B16 cells (mean IC_50_ values of 9.0 and 6.9 μM, respectively; Fig 4C). Furthermore, as observed in Fig 4D and S2 Movie, GM95-UGCG showed typical elisidepsin-induced morphological changes and membrane permeabilization by contrast phase or fluorescence microscopy. Similarly, an increase in cell resistance was observed in HCT-116 parental cells after pretreatment with the UGCG inhibitors D,L-threo-1-Phenyl-2-decanoylamino-3-morpholino-1-propanol (PDMP) and n-Butyldeoxynojyrimicin (*N*B-DNJ, Miglustat hydrochloride) (Fig 5).

**Table 2:** Antiproliferative activity of elisidepsin and other compounds in B16 and GM95 tumor cell lines.

Compound	B16 (IC_50_)^a^	GM95 (IC_50_)^a^	RR^b^
**Elisidepsin**	7.9 x 10^−6^ M	7.0 x 10–5 M	8.9
**Syringomycin E**	1.1 x 10^−5^ M	9.0 x 10^−6^ M	-1.3
**Gemcitabine**	5.6 x 10^−9^ M	8.1 x 10^−9^ M	1.4
**Mitomycin C**	5.7 x 10^−6^ M	2.5 x 10^−6^ M	-2.3
**Paclitaxel**	1.5 x 10^−8^ M	3.4 x 10^−8^ M	2.2
**Cisplatin**	1.8 x 10^−5^ M	9.4 x 10^−6^ M	-2.0
**Doxorubicin**	1.8 x 10^−7^ M	9.4 x 10^−8^ M	-1.9
**Irinotecan**	9.5 x 10^−5^ M	4.5 x 10^−5^ M	-2.1
**Methotrexate**	5.0 x 10^−7^ M	3.7 x 10^−7^ M	-1.4

^a^ IC_50_, cell growth half-maximal inhibitory concentration. Values represent the mean of three different experiments.

^b^ RR: fold change between IC_50_ values from GM95 and B16

## Discussion

In this manuscript, we describe a major role of glycosylceramides in the antitumor effects of elisidepsin. Our results show that elisidepsin specifically interacts with glycosylceramides extracted from human colon carcinoma HCT-116 cells. This lipid fraction was almost absent in an elisidepsin-resistant cell line (HCT-116-Irv). Furthermore, GM95, an UGCG deficient cell line derived from the mouse melanoma B16 cells, was also resistant to elisidepsin. Over-expression of the UGCG gene in these cells reactivated the synthesis of glycosylceramides, rendering them sensitive to elisidepsin at levels comparable to those observed in the parental, UCGC expressing cell line. Altogether, these results indicate that glycosylceramides act as membrane targets of elisidepsin, in order to trigger the membrane permeabilization that leads to drug-induced cell death. These results are not surprising. Given that elisidepsin directly targets the plasma membrane of tumor cells, it was likely that the mechanism of acquired resistance would be related to changes in cell membrane composition. The results exposed here show that, in fact, resistance is principally due to deficiencies in glycosylceramides levels.

For several decades, anticancer drug discovery has been dominated by the idea that proteins were the only differential targets in the cell membrane, whereas lipids were regarded as passive components. Nevertheless, cell membrane lipids have been described as targets for different molecules from varied origin [20–22]. For example, direct interaction between amphotericin B and ergosterol is required for the killing of yeast cells [20]. Similarly, the wide group of defensin peptides include members that bind specifically to phosphoinositides or to glucosylceramides in the cell membrane and induce cell lysis in fungal and tumor cells [23–25]. Our results show that a similar situation happens with elisidepsin in tumor cells, since the presence of glycosylceramides in the cell membrane is necessary for the cytolytic activity of the compound. Indeed, in HCT-116 parental cells which have detectable levels of glycosylceramides, the accumulation of elisidepsin in the cell membrane is very fast. This coincides with the alteration of cell morphology and physiology, the disruption of membrane integrity and the induction of necrotic cell death. Finally, FRET analysis confirmed that elisidepsin molecules are forming some kind of assemblies throughout the plasma membrane of HCT-116 cells, an effect that precedes the disruption of membrane integrity. These findings are in agreement with previous ones showing that elisidepsin rapidly and irreversibly targets the plasma membrane in breast cancer (SKBR-3, MCF-7 and MDA-MB-453), skin (A431) and cervix (HeLa) tumor cell lines [8, 26, 27]. In contrast, HCT-116-Irv cells have reduced levels of glycosylceramides and thus a reduced accumulation of elisidepsin, even after prolonged exposures. Furthermore, elisidepsin cannot be observed in the plasma membrane of these resistant cells. Consequently, we do not detect any change in membrane conductance or permeability and drug treatment did not induce the characteristic necrotic cell death. Moreover, when comparing the sensitivity of HCT-116-Irv cells to other commonly used anticancer drugs, no significant cross-resistance was observed, independently of their mechanisms of action. This lack of cross-resistance against other chemotherapeutics not only reflects the specificity of the resistance developed by these cells against elisidepsin, but also confirms that elisidepsin has a mode of action different from other common anticancer compounds. Altogether, these results validate the hypothesis of glycosylceramides as the cell membrane target of elisidepsin.

Glycosylceramides are important metabolic intermediates which serve as the starting point in the biosynthesis of a wide variety of glycosphingolipids [28, 29]. In a first glycosylation, UGCG or UDP-galactose:ceramide galactosyltransferase (CGT; EC 2.4.2.62) transfer glucose or galactose from UDP-glucose or UDP-galactose to the 1-hydroxyl group of ceramide, yielding glucosylceramide or galactosylceramide, respectively [28]. From these, more complex glycosylsphingolipids, such as lactosylceramide, globotriaosylceramide, monosialoganglioside and others can be synthesized by incorporation of additional sugar residues by different glycosyltransferases [30]. Besides their essential roles as structural components of the cell membrane, these lipids are known to participate in vital cellular processes such as signal transduction, differentiation, migration, apoptosis, proliferation, senescence, and inflammation [31]. Moreover, they are widely described as binding sites for bacteria, viruses and toxins [22, 32, 33]. Glycosylceramides, as well as other glycosphingolipids, have also been intensively studied in regard to their role in tumor pathology [34]. In tumor cells, they have many different functions related to tumorigenesis, cancer progression and drug resistance [35–37]. For this reason, the manipulation of sphingolipid metabolism is currently being studied as a novel and promising strategy for cancer therapy [38–40].

Of note, levels of glycosylceramides and other glycosphingolipids as well as enzymes involved in their metabolism are increased in cancer cells [34, 41]. It has been found that increased expression of UGCG is correlated to the progression of breast cancer, renal cancer, ovarian cancer and leukemia and is frequently correlated with MDR1 levels in tumor samples [42–46]. On the other hand, high mRNA expression of ceramide galactosyltransferase is related to increasing risk of metastases in prostate or breast tumors [47, 48]. Moreover, galactoceramide levels can be used as a molecular marker in human oligodendrogliomas and astrocytomas [49]. Transcriptional repression of the galactocerebrosidase (GALC; EC 3.2.1.46) gene was reported in patients with head and neck squamous cell carcinoma [50]. These reported differences in membrane composition of cancer cells could be extremely important for the activity of elisidepsin since our results show that levels of glycosylceramides determine the response to elisidepsin in tumor cells. Whether analysis of glycosylceramide levels or enzymes involved in its metabolism could be potentially used to select patients that would benefit from elisidepsin therapy remains to be confirmed.

In summary, to exert its antitumor activity, elisidepsin binds to glycosylceramides in the cell membrane. The lack of these lipid species in the plasma membrane confers specific resistance to the drug through lower accumulation on it and thus, to the absence of the biological effects that are normally induced by drug treatment (e.g., changes in membrane conductance, changes in membrane permeability and necrotic cell death). These results highlight the importance of lipid composition and membrane conformation for the sensitivity to elisidepsin. They also indicate that cell membrane lipids are a plausible target for antineoplastic therapy.

## Supporting Information

S1 Movie Plasma membrane permeabilization induced by elisidepsin in HCT-116 cells. Representative sequence of images from HCT-116 and HCT-116-Irv cells exposed to elisidepsin 10 μM for 5 min. In the presence of propidium iodide, undamaged cells show intact nuclei while permeabilized cells show PI stained nuclei.
(AVI)

S2 Movie Plasma membrane permeabilization induced by elisidepsin in GM95-UGCG cells. Representative sequence of images from GM95 and GM95-UGCG cells exposed to elisidepsin 20 μM for 5 min. In the presence of propidium iodide, undamaged cells show intact nuclei while permeabilized cells show PI stained nuclei.
(AVI)
